# Esophageal heating characteristics of very high-power short duration radiofrequency ablation in a myocardial phantom model

**DOI:** 10.1007/s10840-026-02318-6

**Published:** 2026-04-16

**Authors:** Shayekh Abedin, Xingzhou Liu, Amir H. Pour, Michael A. Barry, Stuart P. Thomas, Pierre C. Qian

**Affiliations:** 1 Department of Cardiology, Westmead Hospital, Cnr of Hawkesbury and Darcy Road, Westmead, Sydney, NSW 2145 Australia; 2 Westmead Applied Research Centre, Faculty of Medicine and Health, University of Sydney, Sydney, Australia

**Keywords:** Atrial fibrillation, Radiofrequency ablation, Very high power short duration, Esophageal injury, Esophageal temperature, Ablation biophysics

## Abstract

**Background:**

Very high short duration radiofrequency catheter ablation can rapidly achieve pulmonary vein isolation, however safety to the esophagus during posterior left atrial ablation is uncertain. This study looked to characterise lesion formation and esophageal thermal effects with very high-power short duration (vHPSD), and to compare it with high power short duration (HPSD) and low power long duration (LPLD) radiofrequency ablation, using esophageal heating and esophageal temperature probe (ETP) measurements.

**Methods:**

A thermochromic gel phantom model of posterior left atrial wall with an embedded ETP was used to assess heating kinetics during radiofrequency ablation (RFA). RFA was performed at 30W/20s (LPLD), 50W/5s (HPSD), 90W/3 and 90W/4s (vHPSD). Continuous ETP temperature was recorded, and thermal mapping of esophageal temperature was performed.

**Results:**

90W/3s (5.17 ± 0.01 mm) and 90W/4s (5.62 ± 0.06 mm) produced broader lesions than 50W/5s (4.17 ± 0.11 mm, *p* < 0.0001) and 30W/20 s (3.95 ± 0.38 mm, *p* < 0.01), with similar depth. The ETP underestimated the peak esophageal temperature across all ablation settings with the highest temperature disparity in 30W/20s (3.70 ± 0.60 °C, *p* = 0.0007), 90W/3s (2.87 ± 0.06 °C, *p* < 0.0001) and 90W/4s (2.53 ± 0.25 °C, *p* = 0.0016) groups. Thermal latency was greatest in 90W/4s, followed by 90W/3s, 50W/5s and 30W/20s. Consecutive applications of RFA energy led to heat accumulation and greater esophageal heating, most marked with 90W/4s (41.13 ± 0.12 °C), and 30W/20s (40.90 ± 0.10 °C).

**Conclusion:**

vHPSD produces broader lesions with comparable depth which may result in improved PVI lesion contiguity, however, repeated successive applications with vHPSD may lead to greater esophageal heating and inaccurate ETP monitoring due to temperature disparities and thermal latency.

**Graphical Abstract:**

**Figure:**
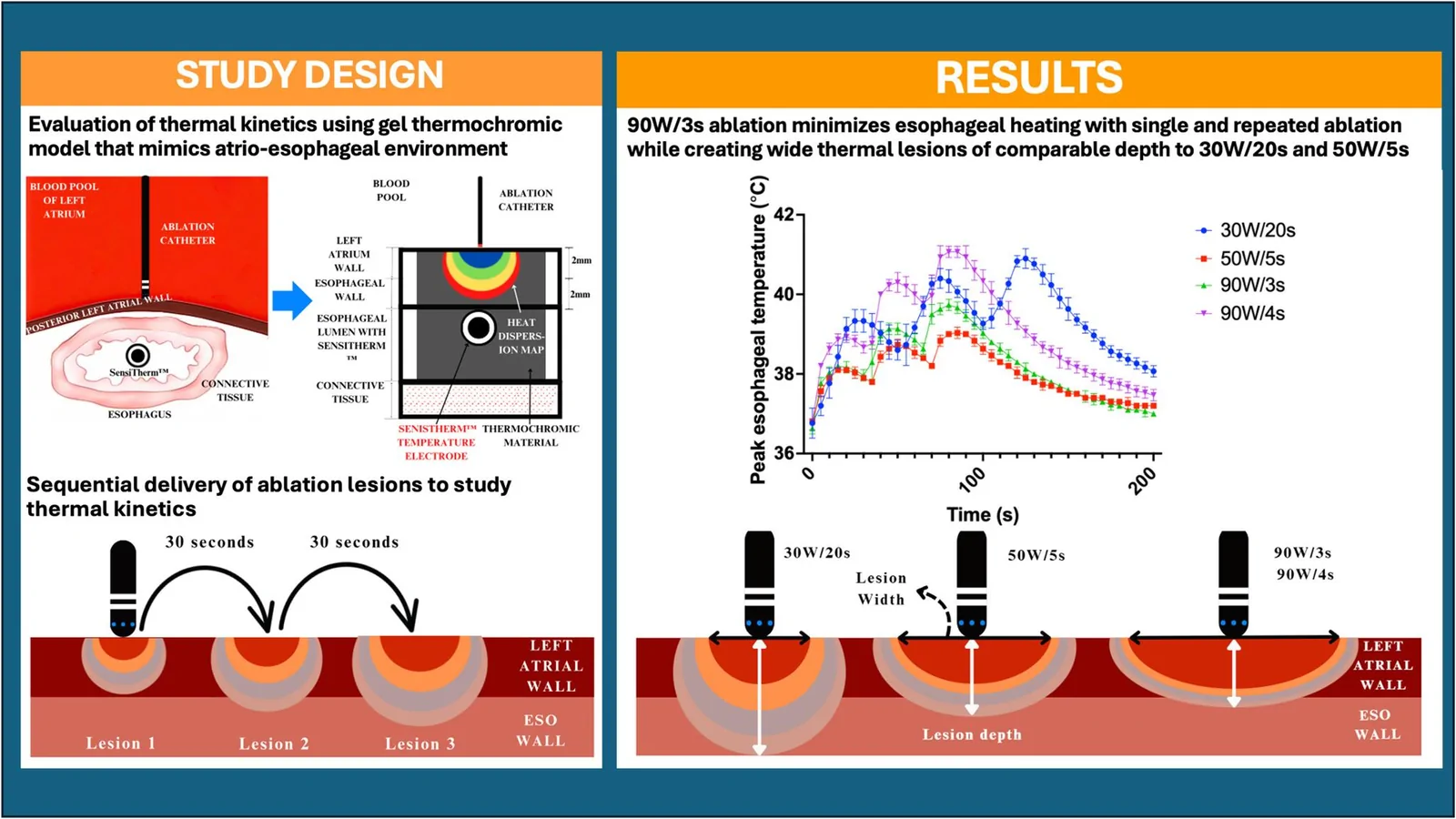

**Supplementary Information:**

The online version contains supplementary material available at https://doi.org/10.1007/s10840-026-02318-6.

## Introduction

Pulmonary vein isolation (PVI) using radiofrequency catheter ablation (RFA) remains a mainstay treatment for atrial fibrillation. Achieving wide antral ablation and isolation of the pulmonary veins can be hampered when ablating posteriorly due to the close anatomical proximity to the esophagus. High power short duration (HPSD) ablation has seen increasing clinical adoption due biophysical advantages in lesion contiguity and minimising lesion depth by reducing conductive heating [1, 2]. However, HPSD ablation has the potential to lead significant esophageal temperature rise and injury similar to LPLD clinically [3, 4]. More recently, very high power-short duration (vHPSD) catheter (QDOT-MICRO™, Biosense Webster, Irvine, CA, USA) has been shown to produce high rates of first pass isolation and reductions in ablation time [5], however vHPSD ablation effects on esophageal heating kinetics has not been well characterised.

Endoscopically detected esophageal injury remains common when routine endoscopy is performed, and the risk is correlated to peak esophageal temperature [6, 7]. Since vHPSD settings essentially precludes useful esophageal temperature probe (ETP) feedback in terminating ablation due to the very short time for energy application and latency in ETP temperature rise, an appreciation of vHSPD ablation biophysics is essential for the operator.

The objective of this study was to compare vHPSD to HPSD and low power long duration (LPLD) in lesion dimensions, heating kinetics, and disparities in esophageal tissue temperature and ETP recorded temperatures after single and repeated ablation applications to provide biophysical principles that may guide use of vHPSD settings near the esophagus.

## Methods

### Myocardial phantom model

A block of radiofrequency myocardial equivalent phantom material [8] was used to approximate a transverse section of the posterior left atrial and adjacent esophageal wall. The phantom material contained 1.5% w/v agar gellan powder substitute (Phytagel, Sigma-Aldrich, P8169) and saline and 30% normal saline in deionized water (i.e., a 0.27% NaCl solution), mimicking the same impedance as myocardial muscle at ablation frequencies (500 kHz). Within this transparent gel, a custom thermochromic liquid crystal (TLC) sheet to be embedded that displayed a gradual transition of colours for temperatures 40 °C and 58 °C. An esophageal temperature probe, SensiTherm™ (Abbott Industries), was placed within a cutout hole in the TLC, set 4 mm away from the gel surface as the esophagus and left atrial wall were each taken to be approximately 2 mm in thickness [9, 10]. To mimic luminal endocardial blood flow, normal saline (0.9%) at 37 °C was perfused over the surface of the gel at a velocity of 55 mm/s [11].

## Delivery of catheter ablation lesions

A 3.5 mm irrigated radiofrequency ablation catheter (Thermocool ^®^ Surround Flow; Biosense Webster, Inc, Diamond Bar, CA) was positioned perpendicular to the TLC sheet. Contact force was not measured, but the model ensured uniform electrode surface contact through positional accuracy. Ablations were delivered using low power, long duration ablation (LPLD) at 30W/20s, and high power, short duration ablation (HPSD) at 50W/5s via a Stockert EP Shuttle Generator, at an irrigation rate of 8mL/min for LPLD and 15mL/min for HPSD, in accordance with the manufacturer’s recommendations. A QDOT Micro™ catheter (Biosense Webster, Inc., CA, USA), was used to perform 90W ablations for 3–4 s (vHPSD) using the nGEN Generator at an irrigation rate that was automatically adjusted between 20-30mL/min as per manufacturers recommendations. The baseline impedance was kept to 100 ± 3Ω, to minimise circuit impedance disturbance [12]. Three ablations were performed at each of the above power settings, with lesion dimensions and temperature measured following each ablation as detailed below. To investigate the effect of “heat stacking”, three ablation cycles were performed with a fixed intermission time of 30 s between each cycle at each power setting. This inter-lesion time was chosen to reflect clinical practice of waiting for the peak esophageal temperature on the ETP to reduce before delivering another nearby lesion.

## Thermal lesion and esophageal temperature measurement

Digital photography of the TLC sheet was performed every 5 s for both HPSD and LPLD, and every 1 s for vHPSD, using a Canon 5DmkII (Canon, Inc., Japan) and macro lens (USM EF 100 mm, Canon, Japan) enabling a high resolution thermal mapping ∼90 pixels/mm with temperature accuracy within 0.5 °C on image analyses via in-house software as previously described [8]. The 53 °C isotherm was defined as the myocardial lesion boundary; lesion width and depth were measured with digital calipers, and lesion volume was calculated using the half-ellipsoid volume formula V = (2/3) x π x d x (w/2)^2^ where V= volume (mm^3^), w = width (mm), d = depth (mm).

During the ablations, SensiTherm™ temperature was continuously recorded via a separate camera (Samsung A52 5G Phone Camera) focused on the SensiTherm™ console, as direct logging of data from the console was not feasible, and then manually transcribed at 1 s intervals.

## Calculating temperature disparity and thermal latency

The thermochrome esophageal temperature was measured at a specific reference point, 0.5 mm upwards from the SensiTherm™ - electrode interface, on the TLC. The temperature at 0.5 mm above the probe was selected as a standard measure to approximate the temperature of the esophageal wall. This specific measurement point was chosen to provide a consistent and relevant indication of thermal impact on the esophageal tissue. Thermal latency for each ablation setting was characterised by late temperature rise (ΔLTR) and Time to T_peak_ (TTP). ΔLTR was defined as the temperature difference between ETP T_peak_ and the RF termination temperature (RTT), i.e. ΔLTR= T_peak_ – RTT. TTP was defined as the time taken to reach ETP T_peak_ following RF ablation cessation.

### Statistical analysis

Statistical analyses were conducted using GraphPad Prism 10.3.1 and R. Descriptive statistics were presented as mean ± SD for continuous variables, and unpaired Student’s t-test, with *p* < 0.05 considered statistically significant.

## Results

### Lesion dimensions

**Fig. 1:**
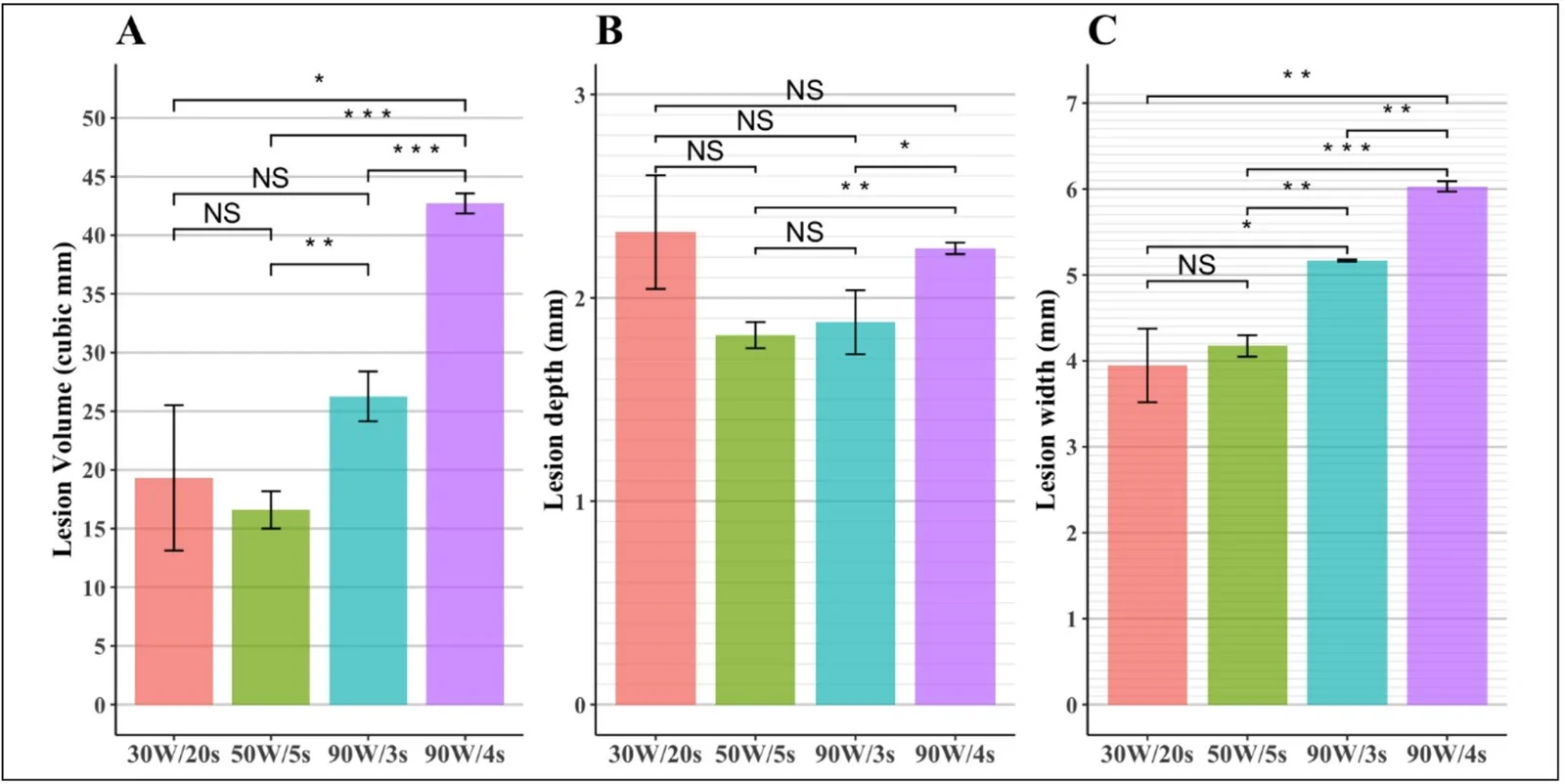
Ablation lesion dimensions across different power settings and duration. (**A)** Comparisons of lesion width, (**B**) comparisons of lesion depth, and (**C**) comparisons of lesion volume across different ablation settings. The major driver of increased lesion volume was an increase in lesion width in the 90W/3s and 90W/4s groups compared to 30W/20s and 50W/5s. Data is presented as mean with 95% confidence intervals. Statistical significance is denoted as follows *p*<0.05 *, *p*<0.01 * *, *p*<0.001 * * *

90W/4s (42.7 ± 0.76 mm^3^) produced the largest lesions by volume compared to 30W/20s (19.3 ± 5.47 mm^3^, *p* < 0.05), 50W/5s (16.6 ± 1.40 mm^3^, *p* < 0.005) and 90W/3s (26.3 ± 1.88 mm^3^, *p* < 0.005, Fig. 1, Table 1). The primary driver of increased lesion volume in 90W/3s and 90W/4s ablations was the increased width of lesions, measuring 5.17 ± 0.01 and 6.03 ± 0.05 mm, respectively, compared to 50W/5s (4.17 ± 0.11 mm, *p* < 0.0001) and 30W/20s (3.95 ± 0.38 mm, *p* < 0.01). 30W/20s ablations produced similar depth to 90W/4 ablations (2.32 ± 0.25 mm vs. 2.24 ± 0.03, *p* = 0.63). In comparison to 30W/20s, lesion depth across the other ablation settings was similar, 50W/5s (1.82 ± 0.06, *p* = 0.06) and 90W/3s (1.88 ± 0.14, *p* = 0.07), showing no statistically significant difference. No steam pops were observed during ablations.

**Table 1:** Quantitation of ablation lesion dimensions at differing RF ablation power and settings

	30W/20s	50W/5s	90W/3s	90W/4s
Lesion depth (mm)	2.32 ± 0.25	1.82 ± 0.06	1.88 ± 0.14	2.24 ± 0.03
Lesion width (mm)	3.95 ± 0.38	4.17 ± 0.11	5.17 ± 0.01*	6.03 ± 0.05*
Lesion volume (mm)	19.3 ± 5.47	16.6 ± 1.40	26.3 ± 1.88	42.7 ± 0.76*

Values are displayed in mean ± SD. Values marked with * are significantly greater (*p* < 0.05) when compared to 30W/20 s lesion dimensions. Lesion volume was largest in the 90W/4s ablations. For 90W/3s and 90W/4s ablations, increased lesion width resulted in increased lesion volume

## Esophageal heating and temperature disparity

**Fig. 2:**
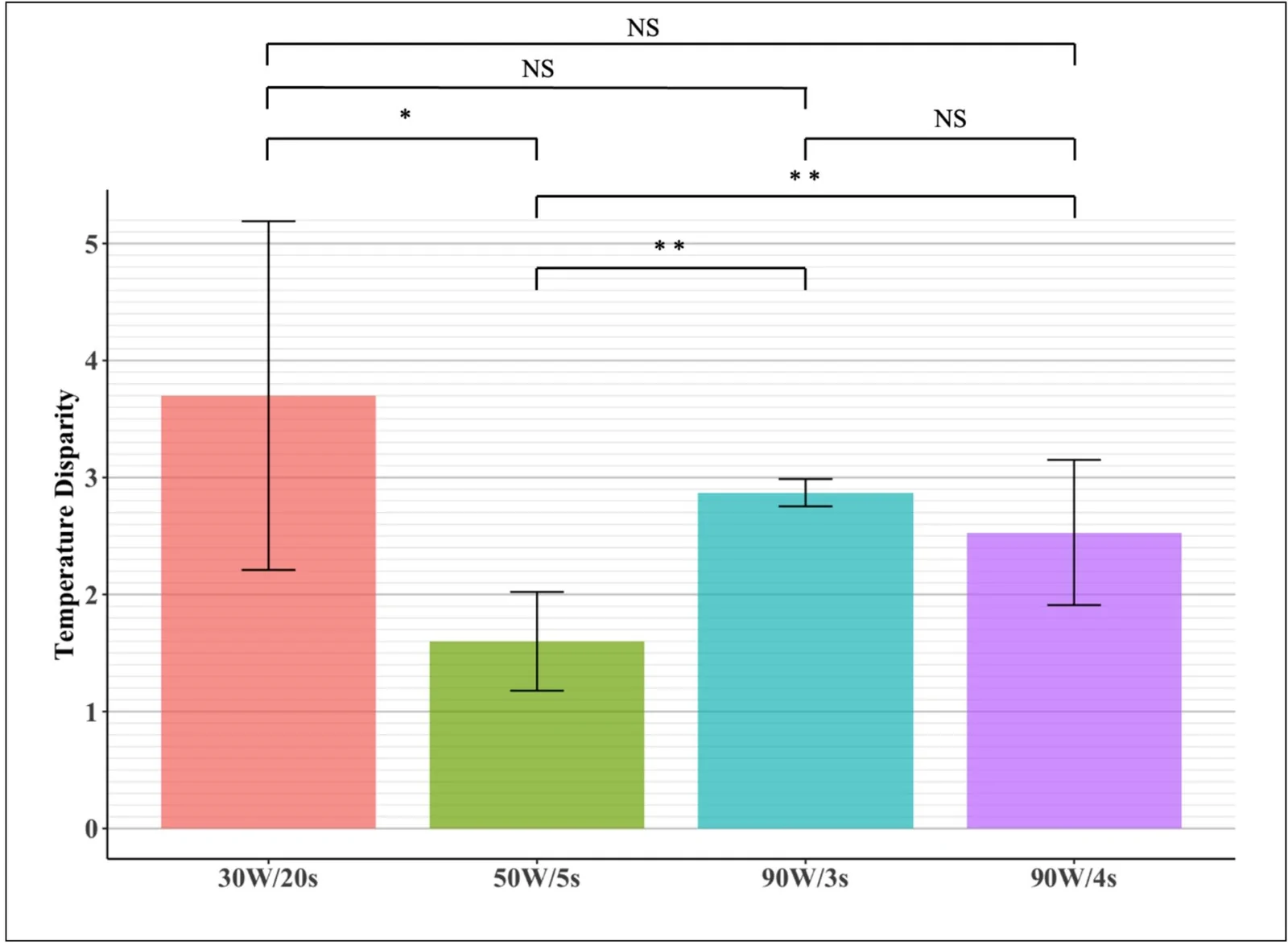
Temperature disparity values across the ablation settings. Temperature disparity was defined as the difference between the thermochrome derived temperature and the esophageal temperature probe (ETP) readings. There was underestimation of the temperature recorded by the ETP across all ablation settings. Data is displayed in mean± confidence intervals. Statistical significance is denoted as follows. *p*<0.05 *, *p*<0.01 * *

ETP T_peak_ was highest in the 30W/20s following a single ablation, followed by the 90W/4s group of ablations. 50W/5s and 90W/3s ablations resulted in the lowest ETP T_peak_ (Table 2). Underestimation of true esophageal temperature by esophageal temperature probes was noted across all ablation power settings (Fig. 2, Table 2). The largest temperature disparity (TD) between the ETP T_peak_ and thermochrome T_peak_ of 3.70 ± 0.60 (*p* = 0.0007) was observed in the 30W/20s group. The 2nd largest TDs were demonstrated by the 90W/3s and 90W/4s, with values of 2.87 ± 0.06 (*p* < 0.0001) and 2.53 ± 0.25 (< 0.0001), respectively. The smallest TD was observed in the 50W/5s group (1.60 ± 0.17, *p* = 0.0003).

**Table 2:** Temperature disparity across ablation settings compared

Ablation Setting (W/s)	SensiTherm™ T_peak_, °C (*n* = 3)	Thermochrome T_peak_, °C (*n* = 3)	TD (*n* = 3)	*p*-value (vs. Thermochrome Temperature)
30W/20s	39.2 ± 0.15	42.9 ± 0.67	3.70 ± 0.60	0.0007
50W/5s	38.1 ± 0.06	39.7 ± 0.23	1.60 ± 0.17	0.0003
90W/3s	38.1 ± 0.06	41.0 ± 0.06	2.87 ± 0.06	< 0.0001
90W/4s	38.9 ± 0.00	41.4 ± 0.57	2.53 ± 0.25	0.0016

The temperature disparity (TD) across all ablation settings was observed, with error bars indicating the standard deviation (± SD). Notably, for all ablation settings, there was an underestimation of peak recorded esophageal temperature (T_peak_)

### Thermal latency and heat stacking

**Fig. 3:**
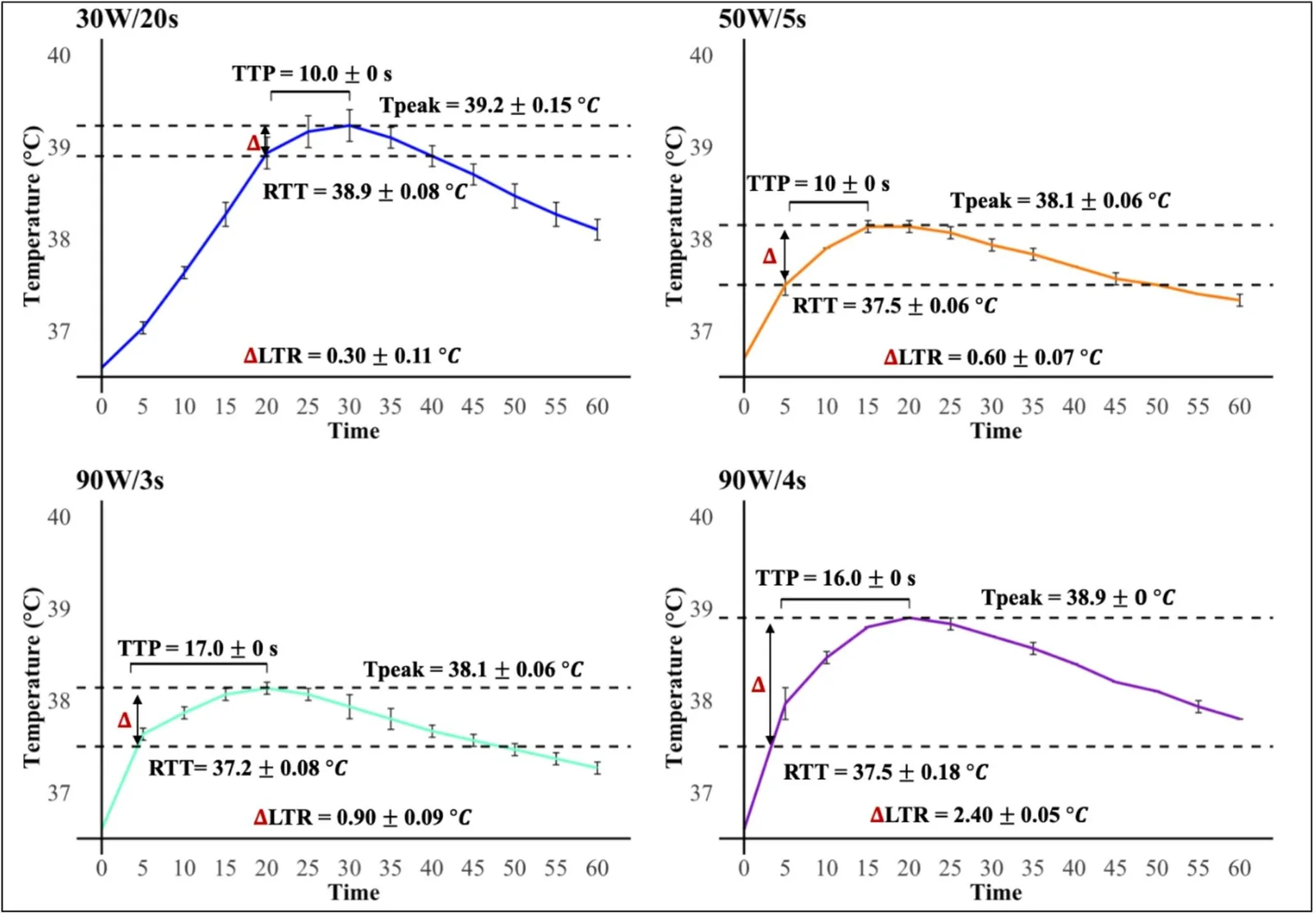
Thermal latency and delayed rise in temperature across varying ablation power and duration. Comparison of esophageal temperature probe (ETP) derived temperature recordings across all ablation settings, in combination with its associated thermodynamic profile containing the recorded esophageal temperature when the ablation was terminated (RTT), peak recorded esophageal temperature (T_peak_), time to reach T_peak_ (TTP) and late temperature rise as the difference in temperature between T_peak_ and RTT (∆LTR). 90W/3s and 90W/4s were associated with a greater extent of tissue heating that persisted for a longer duration following cessation of ablation energy compared to the other ablation settings. T_peak_ was highest in the 90W/4s and 30W/20s groups. Data is presented as mean±SD

90W/4s showed the greatest propensity for heat stacking reaching a T_peak_ of 41.13 ± 0.12 °C, similar to 30W/20s ablation (40.90 ± 0.10 °C, *p* = 0.0572) (Central illustration). In comparison to the 90W/3 seconds, the 50W/5s achieved a lower T_peak_ (39.73 ± 0.06 °C vs. 39.03 ± 0.06 °C, *p* = 0.0001). 50W/5s seconds produced the lowest amount of heat stacking compared to other power setting. ΔLTR was highest in the 90W/4s (2.40 ± 0.05 °C) followed by 90W/3s (0.90 ± 0.09 °C, *p* = 0.0001), 50W/5s (0.60 ± 0.07 °C, *p* < 0.0001) and 30W/20s (0.30 ± 0.11 °C, *p* < 0.0001) groups (Table 3, Fig. 3). The time taken to reach peak temperature following cessation of ablation was highest in the 90W/4s (16 ± 0 s) and 90W/3s (17 ± 0 s) groups and comparatively lower in the 30W/20s (10 ± 0 s) and 50W/5s (10 ± 0) groups (Fig. 3).

**Figure:**
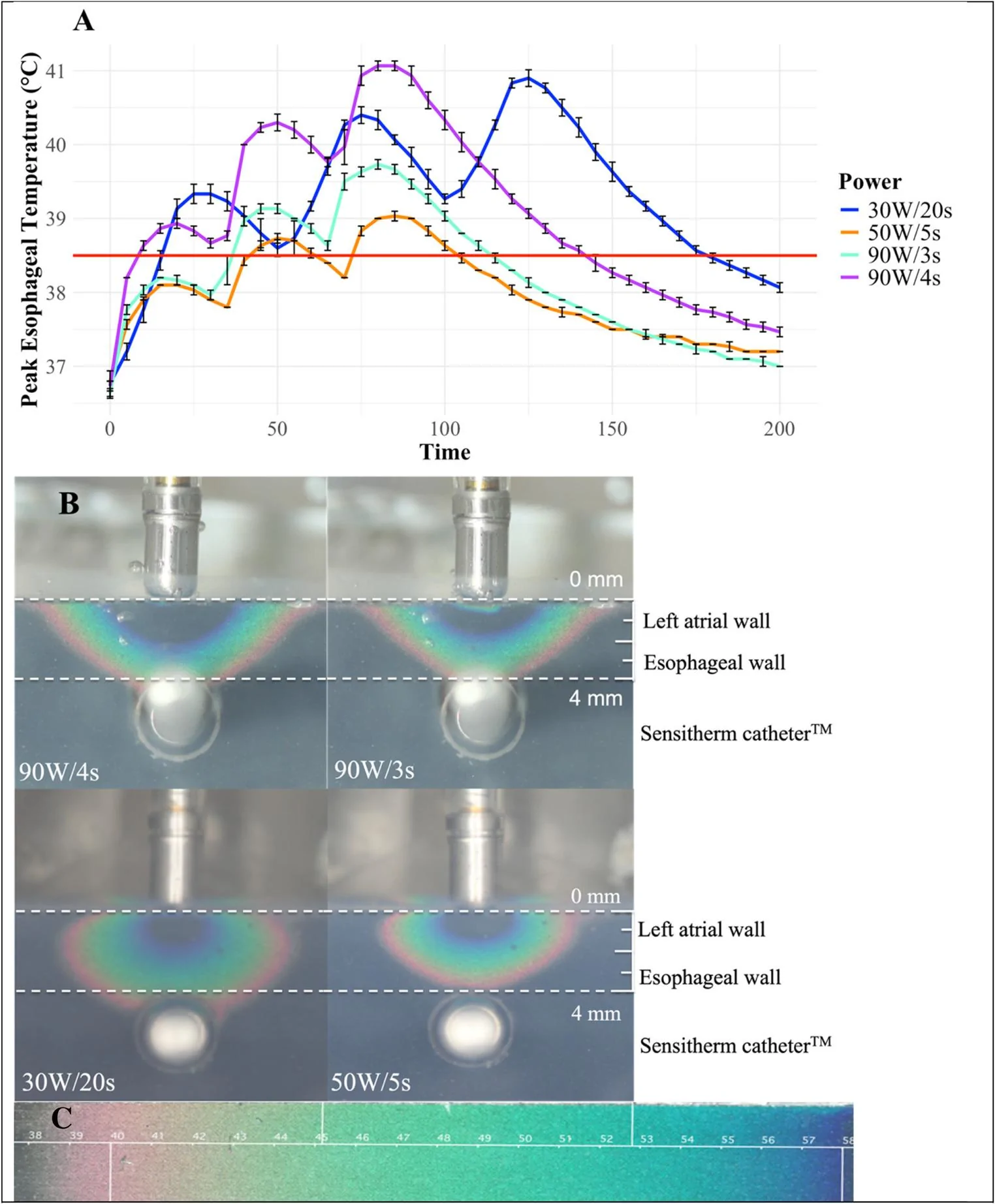
Central illustration: Lesion characteristics and propensity for thermal latency and heat stacking with consecutive ablations at differing RF ablation power and duration. **A** Thermodynamic profiles showing cumulative effects of heat stacking at each ablation setting when consecutive lesion are delivered 30 seconds apart. The mean peak esophageal temperature measured by esophageal temperature probe after each ablation (T_peak_) with error bars (±SD) is depicted. The solid red line indicates a temperature of 38.5°C. Among the above ablation settings, 90W/4s and 30W/20s had the greatest susceptibility to heat stacking reaching the highest esophageal temperatures following multiple consecutive lesions. **B** The thermal lesion was visualised on a thermochromic material with range 40°C to 58°C, with color and corresponding temperature bar depicted in (**C**). 90W/3s and 90W/4s achieved a much broader ablation footprint at the endocardial surface while still maintaining similar lesion depth to 50W/5s and 30W/20s

**Table 3:** Heat stacking across different ablation power and duration settings

Ablation Setting (W/s)	Cycle	Tpeak (°C)	*p*-value (vs. Cycle 1)
30W/20s	Cycle 1	39.33 ± 0.12	N/A
	Cycle 2	40.40 ± 0.10	0.0003
	Cycle 3	40.90 ± 0.10	< 0.0001
50W/5s	Cycle 1	38.10 ± 0.00	N/A
	Cycle 2	38.73 ± 0.06	< 0.0001
	Cycle 3	39.03 ± 0.06	< 0.0001
90W/3s	Cycle 1	38.20 ± 0.00	N/A
	Cycle 2	39.17 ± 0.06	< 0.0001
	Cycle 3	39.73 ± 0.06	< 0.0001
90W/4s	Cycle 1	38.93 ± 0.06	N/A
	Cycle 2	40.30 ± 0.10	< 0.0001
	Cycle 3	41.13 ± 0.12	< 0.0001

Comparing the peak recorded esophageal temperature (Tpeak) across each ablation cycle and across the different ablation regimes, with an unpaired t-test performed, compared to the Cycle 1 Tpeak for each ablation setting. Accumulation of heating occurred across all ablation settings with no restitution of temperature to baseline values during the 30 s waiting period between ablations. The highest propensity for heat stacking, reflected by the largest temperature rise, was associated with the 90W/4s and 30W/20s groups

## Discussion

This is the first study to characterise esophageal heating kinetics with vHPSD ablation with high spatial and temporal resolution. Our main findings were: (1) vHPSD produced wider lesions compared to HPSD and LPLD settings with comparable depth, (2) 90W/4s and 90W/3s results in higher disparity between measured and true esophageal temperatures and, after cessation of ablation energy, higher peaks with longer lag time on recorded ETP temperatures, and (3) heat stacking was more pronounced with 90W/4s and 30W/20s while 90W/3s or 50W/5s produced the least esophageal heating with single and repeated ablations.

### vHPSD produced wider lesions

vHPSD using 90W/3s and 90W/4s achieved similar depth to 50W/5s and 30W/20s but with dramatically greater lesion width and greater lesion volumes, consistent with animal studies [1]. A LPLD setting 20–25 W with low irrigation flow demonstrated a similar lesion geometry to vHPSD with less surface sparing and greater transmurality [13]. The lesion geometry using 90W/3s and 90W/4s resembled that of non-irrigated ablation lesions, indicating that the 90W power was sufficient to overcome surface convective cooling from catheter irrigation and blood flow to create lesions with broad endocardial footprints. This may improve lesion-to-lesion consistency and overlap, suggested by higher rates of first pass PVI in recent clinical trials with a HPSD and vHPSD approach, with the added benefit of a reduction in procedural time [14–16]. The geometric profile of vHPSD lesions has been shown to be effective in ablating other thin walled structures such as the right ventricular outflow tract where it has exhibited high acute success in treating pre-ventricular ectopics [17]. However, there are concerns of increased stroke risk with HPSD settings particularly when shorter interlesion distances are used which may be due to the larger endocardial footprint of the lesion [18].

### Esophageal heating is minimised by 90W for 3s and avoiding heat stacking

Esophageal heating using 50W/5s and 90W/3s was lower than 30W/20s and 90W/4s, which can be explained by the overall smaller joulage (power x duration) delivered. Lower joulage delivered in the vicinity of the esophagus should result in less conductive heating and esophageal injury, which is supported by clinical trials showing that HPSD produced lower or similar rates of esophageal injury LPLD [16, 19]. However, when repeated applications of vHPSD are delivered in close vicinity, caution should be exercised as the rate of heat flux (joules/time) is relatively increased when the same interlesion cooling period is used. While 90W/4s delivered significantly less joules than 30W/20s, as the same waiting period of 30s was used, 3 × 90W lesions delivered approximately 360 J over 72s (5 J/s), identical to 3 × 30W ablations which delivered 600 J over 120s (5 J/s). When the same average rate of radiofrequency energy flux is calculated for 50W/5s and 90W/3s, it is substantially lower at 3.3 J/s and 3.9 J/s respectively. The higher rate of energy delivery over the span of 3 lesions rather than the power used was the determinant in the peak temperature at the esophagus. Therefore vHPSD ablation and HPSD can be safely delivered near the esophagus when limited to single ablations, and during linear ablation ensuring sufficient interlesion distances and interlesion times, or using an interrupted technique [20], to enable time for esophageal heat dissipation. Even in the era of pulsed field ablation (PFA), there have been significant challenges in attaining long term durability using 1st generation PFA catheters for linear ablation outside of the pulmonary veins in anatomical areas adjacent to the esophagus, as well as concerns regarding the safety of PFA when delivered in close proximity to the coronary arteries [21, 22]. Therefore, a strong understanding of vHPSD and HPSD biophysics remains relevant in the management of complex arrhythmias.

### Lower accuracy of esophageal temperature monitoring with vHPSD

The disparity between true and measured esophageal temperature were highest in the LPLD and vHPSD ablations (Fig. 2). The time to detect the peak temperature (TTP) on ETP and delayed rise in temperature (ΔLTR) was greater the higher the power used (Fig. 3), consistent with clinical observations [3, 23]. The use of ETP to monitor temperature suffers from the need for the thermocouple to be within 2 cm of the ablation catheter to accurately detect increases in luminal esophageal temperature [9, 24]. With vHPSD and HPSD, even with ETP placed directly below the ablation site, as in our model, most of the recorded temperature rise occurs after ablation is terminated (Fig. 3). In particular, it took vHPSD ablations ≥ 16s to peak in temperature after a 4s delivery. Therefore, relying on ETP temperature for guiding when to cease energy delivery is inherently problematic when HPSD or vHPSD settings are used. Rather, ETPs may be more useful at these power settings to monitor average temperature trends to confirm the adequacy of employed prophylactic ablation strategies in limiting esophageal heating.

### Limitations

Our biophysical investigation used an in vitro phantom model to ascertain heating kinetics to demonstrate biophysical principles with strength in its temporal and spatial resolution and reproducibility. This enables comparisons of ablation settings in the same highly controlled environment. This study has several important limitations. Firstly, this model is unable to inherently replicate the complex layered anatomy of biological tissue including the posterior left atrial wall, pericardial fat and esophageal mucosa. However, the biophysical and thermodynamic patterns observed appeared to be concordant with existing preclinical and clinical data. Secondly, contact force was not measured. Due to the non-deformable nature of the TLC, increasing applied pressure would not alter catheter deformation or contact. Hence, small variations in contact force are unlikely to impact the results obtained from this experimental model.

## Conclusion

We demonstrated 90W/3s settings can minimise esophageal heating both with single and repeated ablation while creating wide thermal lesions of comparable depth to 50W/5s and 30W/20s. Using vHPSD and HPSD setting, ETP recordings do not provide an accurate real time reflection of actual esophageal temperature and should not be relied upon to curtail ablation. Instead, empiric dosing of ablation energy should be performed using vHPSD or HPSD and sufficient cooldown time given to minimise esophageal thermal injury.

## Supplementary Information

Below is the link to the electronic supplementary material.

Supplementary Material 1.

## Data Availability

The data supporting the findings of this study are available from the corresponding author upon reasonable request.

## Abbreviations

HPSD: High Power, Short Duration Ablation
vHPSD: Very High Power, Short Duration Ablation
LPLD: Low Power, Long Duration
RFA: Radiofrequency Catheter Ablation
ETP: Esophageal Temperature Probe
PVI: Pulmonary Vein Isolation
TLC: Thermochromic Liquid Crystal
TD: Temperature disparity
T_peak_: Peak Esophageal Temperature
TTP: Time to peak temperature following ablation cessation
RTT: Radiofrequency ablation termination temperature
ΔLTR: Late temperature rise = RTT-T_peak_
PFA: Pulsed Field Ablation
